# Endoscopic Resection for Duodenal Neuroendocrine Neoplasms between 10 and 20 mm—A Systematic Review and Meta-Analysis

**DOI:** 10.3390/jcm13051466

**Published:** 2024-03-02

**Authors:** Roberta Elisa Rossi, Benedetta Masoni, Sara Massironi, Giacomo Marcozzi, Gianluca Franchellucci, Angelo Zullo, Antonio Facciorusso, Silvia Carrara, Nadim Mahmud, Giulia Migliorisi, Silvia Ferretti, Roberta Maselli, Cesare Hassan, Alessandro Repici

**Affiliations:** 1 Gastroenterology and Endoscopy Unit, IRCCS Humanitas Research Hospital, Via Manzoni 56, Rozzano, 20089 Milan, Italy; benedetta.masoni@humanitas.it (B.M.); giacomo.marcozzi@humanitas.it (G.M.); gianluca.franchellucci@humanitas.it (G.F.); silvia.carrara@humanitas.it (S.C.); giulia.migliorisi@humanitas.it (G.M.); silvia.ferretti@humanitas.it (S.F.); roberta.maselli@hunimed.eu (R.M.); cesare.hassan@hunimed.eu (C.H.); alessandro.repici@hunimed.eu (A.R.); 2 Department of Biomedical Sciences, Humanitas University, Via Rita Levi Montalcini 4, Pieve Emanuele, 20072 Milan, Italy; 3 Division of Gastroenterology and Center for Autoimmune Liver Diseases, Fondazione IRCCS San Gerardo dei Tintori, Department of Medicine and Surgery, University of Milano-Bicocca, 20900 Monza, Italy; sara.massironi@libero.it; 4 Gastroenterology Unit, ‘Nuovo Regina Margherita’ Hospital, 00153 Rome, Italy; zullo66@gmail.com; 5 Section of Gastroenterology, Department of Medical Sciences, University of Foggia, 71122 Foggia, Italy; antonio.facciorusso@unifg.it; 6 Division of Gastroenterology and Hepatology, University of Pennsylvania Perelman School of Medicine, Philadelphia, PA 19104, USA; nadim.mahmud@pennmedicine.upenn.edu

**Keywords:** neuroendocrine neoplasms, duodenal neuroendocrine neoplasms, treatment, endoscopic submucosal dissection, endoscopic mucosal resection

## Abstract

**Background.** The optimal management of duodenal neuroendocrine neoplasms (dNENs) sized 10–20 mm remains controversial and although endoscopic resection is increasingly performed instead of surgery, the therapeutic approach in this setting is not fully standardized. We performed a systematic review of the literature and a meta-analysis to clarify the outcomes of endoscopic resection for 10–20 mm dNENs in terms of efficacy (i.e., recurrence rate) and safety. **Methods.** A computerized literature search was performed using relevant keywords to identify pertinent articles published until January 2023. **Results.** Seven retrospective studies were included in this systematic review. The overall recurrence rate was 14.6% (95%CI 5.4–27.4) in 65 patients analyzed, without significant heterogeneity. When considering studies specifically focused on endoscopic mucosal resection, the recurrence rate was 20.5% (95%CI 10.7–32.4), without significant heterogeneity. The ability to obtain the free margin after endoscopic resection ranged between 36% and 100%. No complications were observed in the four studies reporting this information. **Conclusions.** Endoscopic resection could be the first treatment option in patients with dNENs sized 10–20 mm and without evidence of metastatic disease. Further studies are needed to draw more solid conclusions, particularly in terms of superiority among the available endoscopic techniques.

## 1. Introduction

The overall incidence of neuroendocrine neoplasms (NENs) has increased in recent decades, with gastroenteropancreatic forms representing the vast majority and accounting for approximately 80% of all NENs [1,2,3].

Duodenal NENs (dNENs) account for about 5% of all the gastrointestinal NENs (GI-NENs). Although emerging environmental factors may play a role in their development, it is still unclear to what extent the increase in their incidence is due to the increased use of diagnostic GI endoscopy worldwide [4]. Even if dNENs have always been considered rare and often indolent entities, they are known to hold a metastatic potential independently of the size of the primary tumor, which can be up to 50% at the time of the first diagnosis [5]. While some prognostic factors for pancreatic, gastric, and small bowel NEN management have been identified, this is not entirely true for the duodenal counterparts. Indeed, the lack of clear evidence on prognostic factors for dNENs and their great heterogeneity in terms of biological behavior has led to a poor understanding of their natural history and to a scarcity of standardized treatment protocols [5,6].

According to previous European Neuroendocrine Tumor Society (ENETs) guidelines [7,8], endoscopic resection is reported to be effective and safe only for non-functioning duodenal lesions measuring <10 mm, confined to the submucosal layer and in the absence of lymph node (LN) metastases. For lesions larger than 20 mm and/or in the presence of infiltration beyond the submucosa (T2 neoplasm) or LN metastases, a surgical resection is advised in order to guarantee a better oncological radicality. Furthermore, recent data [9] reported that conventional and functional imaging is associated with a high risk of understaging of dNENs, suggesting a need to be cautious when deciding on a more conservative approach to this subgroup of tumors and highlighting the need for a proper tumor staging with endoscopic ultrasound (EUS), despite the primary tumor size. In line with recent findings, the new guidelines [8] are even more conservative, suggesting an endoscopic approach for lesions <5 mm and highlighting the need for EUS for ≥5 mm lesions. In between, the treatment of dNENs sized 10–20 mm remains controversial. As it is less invasive than surgery, endoscopic resection is increasingly performed instead of surgery, with promising results, even if this therapeutic approach in such a specific setting is not fully standardized, considering that the majority of available studies are small non-controlled retrospective case series or case reports with no long-term outcomes [9]. Available endoscopic resection methods include endoscopic submucosal dissection (ESD), endoscopic mucosal resection (EMR), EMR with a ligation device (EMR-L), or EMR after circumferential pre-cutting (EMR-P). Among them, ESD allows a higher rate of complete resection, but it owns higher risk of bleeding and perforation and a longer procedure time as compared to the other methods. Which endoscopic technique should be preferred among the others is still not clear, and in the majority of the cases the choice depends on both the tumor’s features and the endoscopist’s expertise [10]. In this grey area, according to the most recent guidelines [8], endoscopic resection might be considered only in selected cases and generally for tumors smaller than 15 mm.

Based on all these observations, we performed a systematic review and meta-analysis of data on efficacy (i.e., recurrence rate) and safety of the endoscopic resection for dNENs sized between 10 and 20 mm, aiming to clarify this still-grey area.

## 2. Materials and Methods

This systematic review was performed following the Preferred Reporting Items for Systematic Reviews and Meta-analysis (PRISMA) recommendations, but was not registered.

A computerized literature search was performed in PubMed using both free language words and phrases and medical subject heading terms including neuroendocrine neoplasms, duodenal neuroendocrine neoplasms, treatment, endoscopic submucosal dissection, and endoscopic mucosal resection, with the search strategy last updated in January 2023. The following terms were used for the literature search: ((“net” [All Fields]) OR (“duodenal net” [All Fields] OR “duodenal neuroendocrine” [All Fields] OR “duodenal neuroendocrine neoplasm” [All Fields] OR “duodenal neuroendocrine neoplasms” [All Fields] OR “duodenal neuroendocrine tumor” [All Fields] OR “duodenal neuroendocrine tumors” [All Fields] OR “duodenal neuroendocrine tumour” [All Fields] OR “duodenal neuroendocrine tumours” [All Fields])) AND ((“endoscopic resection” [All Fields] OR “endoscopic resection methods” [All Fields] OR “endoscopic resection method” [All Fields] OR “endoscopic resection technique” [All Fields] OR “endoscopic result” [All Fields]) OR (“mucosectomy” [All Fields] OR “mucosectomy band” [All Fields] OR “mucosectomy band ablation” [All Fields] OR “mucosectomy patients” [All Fields] OR “mucosectomy polypectomy” [All Fields]) OR (“endoscopic submucosal dissection” [All Fields] OR “endoscopic submucosal dissection for colorectal” [All Fields] OR “endoscopic submucosal dissection resection” [All Fields] OR “endoscopic submucosal dissection for” [All Fields]) OR (“endoscopic mucosal resection” [All Fields])).

All the available primary studies published between 2010 and 2023 were considered, whereas non-English language papers were excluded. Studies considered potentially eligible were retrieved and evaluated as full texts. Disagreements were resolved by consensus.

### 2.1. Eligibility Criteria

The eligible studies investigated the outcomes of endoscopic resection in patients with histologically confirmed dNENs of size 10–20 mm. Both retrospective and prospective studies were included.

There were no restrictions on gender, age, risk factors, or symptoms among the study participants. The type of endoscopic resection included ESD and EMR; modified EMR methods, including EMR-L or EMR-P, could also be included where specified.

### 2.2. Outcome Measures

The primary endpoint was efficacy of endoscopic resection in this subset of patients. Efficacy was evaluated in terms of rate of tumor recurrence. As secondary endpoints we considered the rate of radical resection (i.e., the presence of free margin associated with tumor resection) and the safety of endoscopic resection, which was assessed in terms of complications (i.e., bleeding, perforation, stenosis).

### 2.3. Selection of Studies

Articles were first selected based on an evaluation of titles and abstracts. Then, abstracts that did not meet the inclusion criteria were excluded. The selected articles were extensively reviewed to identify those that fit the defined selection criteria. Duplicate studies were removed. Clinical cases, commentaries, reviews, systematic reviews, meta-analyses, and letters were excluded. A manual reference search of all retrieved studies was also performed to search for additional potential studies for inclusion in the systematic review. Furthermore, authors of selected articles, which did not clearly report separated data on dNENs sized between 10 and 20 mm, were individually contacted via e-mail in order to acquire those missing data.

### 2.4. Methods of the Review of the Literature and Syntesis

Three reviewers (BM, GM, GF) assessed all the studies identified as described above: each paper was re-examined to confirm that it met the inclusion criteria and then ranked for its methodological quality, based on previously reported criteria [11].

Data on types of participants and outcome measures were extracted independently by the reviewers. Only in the case of discrepancies was the further and final judgment of an independent clinical expert (AR, CH, RER) applied. The excluded studies and the reasons for exclusion were recorded. In case of duplicate publications, the most recent version was considered.

To ensure a rigorous and transparent evaluation of the methodological quality and risk of bias in the included studies, we employed the Newcastle-Ottawa Scale (NOS) for cohort studies. Each study was evaluated and categorized based on its total score as follows: 0–3 points indicated low quality, 4–6 points indicated moderate quality, and 7–9 points indicated high quality.

All the studies included were evaluated for their methodological quality, considering their study design (cohort studies or case series), patient selection (consecutive or non-consecutive), data collection (prospective, retrospective, or unknown), and spectrum composition (reflecting, or not, the representativeness of the patients of the clinical practice), according to previously defined standards [11].

Where available, the following information was extracted from each study: total number of patients, study design, median duodenal tumor size, duodenal site, degree of tumor differentiation (i.e., grading according to the WHO 2010 classification system), functioning or non-functioning tumor, type of endoscopic procedure, free margin status, recurrence rate, complications, PFS, and overall survival (OS).

The heterogeneity of the results was assessed in the analysis using the I^2^ statistic, which measures the percentage of total variation across studies that is due to heterogeneity rather than chance. We considered substantial heterogeneity to be present when I^2^ exceeded 50%.

If substantial heterogeneity was present, no meta-analytic syntheses were performed, but only qualitative analysis summarizing the findings of individual studies was done. In case of non-significant heterogeneity, random-effects models were used to synthesize the measure estimates. We employed random-effects models to synthesize the measure estimates, and the pooled effect estimates derived from our meta-analyses were visually presented using Forest plots. The statistical analyses were conducted using MedCalc Statistical Software version 14.8.1 (MedCalc Software bvba, Ostend, Belgium; http://www.medcalc.org; 2014, accessed on 1 December 2023).

## 3. Result

The search strategy produced 339 studies. After removing duplicates, a total of 37 articles were selected by title and abstract. Filtering by article type, 19 studies reporting endoscopic resection data of dNENs were considered, for a total of 105 tumors.

**Figure 1:**
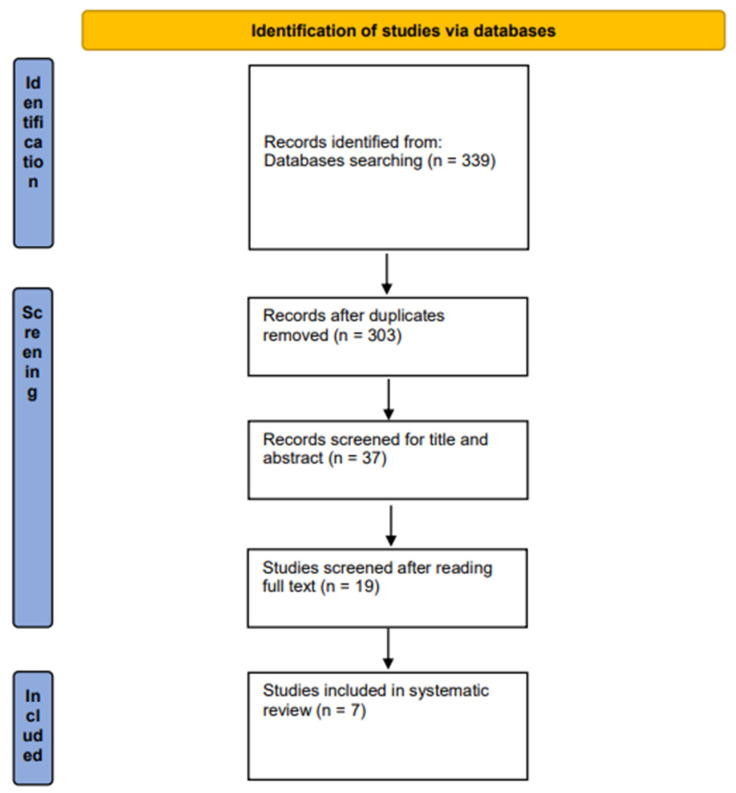
Preferred Reporting Items for Systematic Reviews (PRISMA) flowchart for identified studies on endoscopic resection for duodenal neuroendocrine tumors.

For the purpose of our systematic review, seven studies [5,12,13,14,15,16,17], met the inclusion criteria and contained relevant data on dNENs sized between 10 mm and 20 mm. The other studies were discarded due to insufficient data. Figure 1 represents the study selection process in the PRISMA diagram.

The included studies were all retrospective (Table 1). According to the Newcastle-Ottawa Scale (NOS) for cohort studies, six studies were categorized as moderate quality [5,14,15,16,17,18] and one study only as high quality, with a score of 7/9 [13]. Information regarding the histologic grading was available for only 29 dNENs undergoing endoscopic resection: 21 tumors (72.5%) were grade 1 (G1), 8 (27.5%) were grade 2 (G2), and none were classified as grade 3 (G3).

The study with the largest sample size [14] included 20 dNENs between 10 and 20 mm treated with EMR and concluded that there was no statistically significant difference in progression-free survival (PFS) between surgical resection and EMR.

Overall, we collected accurate information about the treatment of 65 tumors: 59 of them (91%) had been treated with EMR, 5 (8%) with ESD, and 1 (1%) with a cold polypectomy technique. In addition, *en bloc* resection was being practiced in seven out of nine tumors (77.9%) in Mahmud [15], in eight out of nine tumors in Lee’s study (88.9%) [13], and for all the four tumors in Yokoyama’s study (100%) [16], while this information was not reported in the remaining studies.

In terms of follow-up, in the seven included studies, the follow-up ranged from a minimum of 17.5 months [15] to a maximum of 95.7 months [14], with a mean follow-up of 49 months.

**Figure 2:**
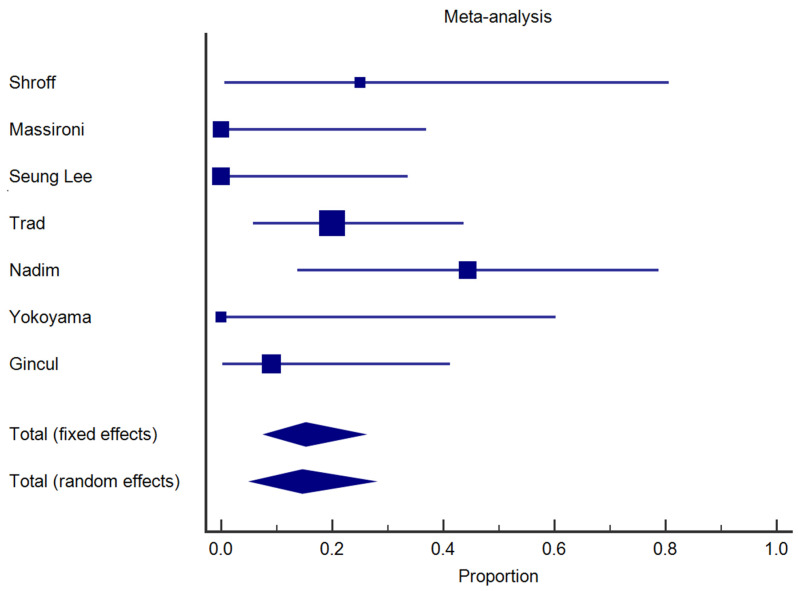
Forest plot illustrating the results of the analysis of the recurrence rate after endoscopic treatment in all the seven included studies.

When considering all the endoscopic techniques together [5,12,13,14,15,16,17], the overall recurrence rate was 14.6% (95%CI 5.4–27.4) in a total of 65 patients analyzed, without significant heterogeneity of the results (I^2^ = 42.8%, *p* = 0.1) (Figure 2). When considering studies specifically focused on EMR techniques [12,14,15,16,17], the recurrence rate was 20.5% (95%CI 10.7–32.4) in a total of 47 patients examined, without significant heterogeneity (I^2^ = 0%, *p* = 0.4). Only two studies [5,13] explored the efficacy and radicality of ESD technique, analyzing a total of only five patients, and neither reported any cases of recurrence.

During the follow-up, Tran’s study reported four cases of local post-EMR recurrence that underwent subsequent surgical resection [14]; three cases of local disease recurrence were reported in Mahmud’s study, with no significant difference in the local recurrence rate between EMR and simple polypectomy [15]. Among the studies considered, only one metastatic lesion to local lymph nodes was observed 6 months after endoscopic resection and the patient underwent complementary surgical treatment [12]. No deaths were observed among the 65 analyzed patients during the follow-up period.

Data on the ability to obtain the free margin (R0) on histopathological examination after endoscopic tumor resection were only available in some of the studies. In Yokoyama’s study, the R0 rate was 100% [16], in Mahmud’s study 77.8% [15], and in Shroff’s study 50% [12]. In Lee’s study, on the other hand, the R0 rate was 0% in the subgroup with lesion size between 10 and 20 mm, in contrast to those in the other two subgroups (1–5 mm, 6–10 mm); lymphovascular invasion also occurred significantly more frequently among those with lesion size ≥11 mm [13].

**Figure 3:**
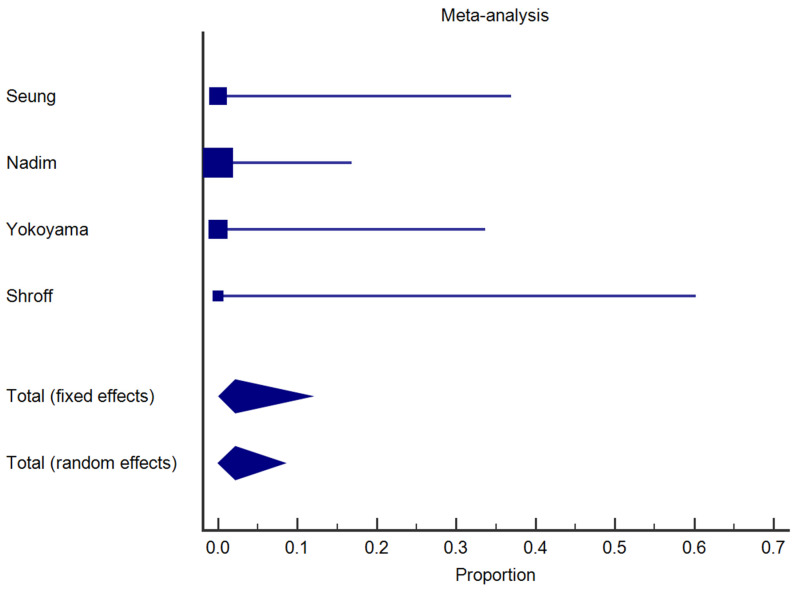
Forest plot illustrating the results of the analysis on the safety of endoscopic treatment in the four studies analyzed. The plot shows proportions with corresponding 95% confidence intervals for each study, indicating the absence of severe complications.

As concerned safety, in the analysis of the examined studies (Figure 3), it was found that no severe complications, such as significant bleeding during or after the procedure, perforation, or stenosis, were reported. 

## 4. Discussion

According to the present systematic review, endoscopic resection could be the first treatment option in patients with dNENs sized 10–20 mm without evidence of metastatic disease, and it might be a valid therapeutic alternative to surgical resection in this subgroup of patients, whose treatment has not yet been fully standardized.

Currently, there is no consensus on the exact relationship between the dNENs’ diameter and the likelihood of LN involvement, and even if the tumor size seems to be a prognostic factor for tumor recurrence [18], recent data proved that dNENs can be metastatic at any size [5]. Direct surgical resection is suggested in the presence of LN metastases, peri-ampullary lesions, or for lesions with a size >20 mm (i.e., high risk of LN involvement), while endoscopic resection is considered for lesions <10 mm as long as they are limited to the submucosal layer (i.e., low risk of LN involvement), if there is no EUS evidence of LN involvement [7,8,19]. Intermediate NENs measuring 10–20 mm still remains a grey area, and their treatment remains often managed on a case-by-case basis depending on the single center’s decision and/or attitude. According to the most recent guidelines [8], a cut-off of 15 mm might be considered for endoscopic resection, but only in a selected subgroup of tumors and provided that EUS shows no LN or vascular involvement.

An EUS evaluation prior to performing any type of resection (endoscopic or surgical) is mandatory and has proven to have a sensitivity as high as 94% in selecting appropriate candidates for endoscopic resection [20], as LN metastases may often be missed during the initial tumor staging using conventional imaging [8,18]. In the studies included in the current systematic review, all the patients included in three of the studies [14,15,16] had undergone EUS prior to endoscopy, while this information was not available for the remaining ones.

According to published data, ESD seems to be superior in terms of oncological radicality, scoring as high as 100% [21] vs. 56% for EMR [22], and in avoiding recurrence when compared to EMR for dNENs, probably due to its larger tissue removal. However, ESD is a technique requiring a longer execution time [23] and significantly higher endoscopic expertise than EMR and may therefore not always be available in non-tertiary centers [21,22,24]. Moreover, since the duodenal wall is normally thinner than its gastric or rectal counterpart, ESD tends to be associated with a higher risk of complications [23,25]. It is worth noting that, when considering dNENs, studies on endoscopic resection techniques are scanty, especially for ESD, and herein only two studies analyzed ESD, for a total of five patients, and no recurrence was observed. However, due to the small sample size, no comparison can be made between the different endoscopic techniques, and further research with a larger number of studies and more comprehensive data is needed to provide a more robust understanding of the recurrence rates associated with different endoscopic techniques.

In the present systematic review, the vast majority of the dNENs examined were removed using EMR, and during a median follow-up of 49 months, eight cases of disease recurrence were reported in two studies only [14,15], with no distant metastatic lesions, which suggests that endoscopic resection can be considered in selected dNENs sized 10–20 mm. According to data from the literature, in a retrospective study including 12 dNEN patients treated with EMR, 34 who had local resections, and 29 who underwent pancreaticoduodenectomy, 11 tumor recurrences were observed with an overall median follow-up of 27 months, and four patients died of their disease [18]. No difference in recurrence rate was identified among the different treatment options, although tumors selected for EMR were smaller than tumors surgically treated, which highlights that tumor size is considered a prognostic factor for tumor recurrence. In line with these data, in the study by Kim et al. [21], no recurrence was observed in 36 lesions endoscopically treated, but these results were influenced by the fact that this study included dNENs ≤ 10 mm only and that the mean follow-up period was as short as 17 months.

Unfortunately, data regarding the radicality of endoscopic resection were scarce, available in only a few studies but as high as 100% in Yokoyama’s study [16] and 77.8% in Mahmud [15]. In the French retrospective study by Gincul et al., a positive margin rate (R1) as high as 44% was found and, in particular, in the univariate analysis, no significant differences were reported between the resection techniques (EMR/EMR-C) or tumor sizes (whether <10 mm or 10–20 mm), suggesting that the major role in oncological radicality may be played by the resection technique itself instead of by the tumor size [17]. However, as recently reported, when dNENs < 20 mm are considered, the actual impact of a positive margin status itself (R1) on the risk of recurrence after endoscopic resection has yet to be clarified, since data regarding the statistical significance of this association seem to be contradictory among different studies [10,26,27].

Regarding safety, endoscopic resection appears to be a safe technique. In the current analysis, no complications were reported. However, this favorable outcome can be attributed to the limited number of patients enrolled in each study, which may have helped to mitigate the occurrence of adverse events.

The major limitation of the present study is definitely the limited sample size. Aside from the relative rarity of this disease, the main reason for this has been the paucity of data regarding sole endoscopic resections of 10–20 mm dNENs in the literature, since often for this subset of lesions the surgical choice prevails over the endoscopic alternative due to the fear of micro-metastases, the patients’ preferences, or the lack of endoscopic expertise, leading to a rather small pool of data for us to begin with. Moreover, the vast majority of the studies available in the literature that focused on endoscopic resection included almost exclusively smaller dNENs (<10 mm), since their treatment with the endoscopic approach is standardized at the present time, while separate data regarding dNENs measuring 10–20 mm were available in only seven studies, providing a small sample size. Of note, the analyzed studies included mainly tumors <15 mm (see Table 1), which does not allow us to draw solid conclusions regarding dNENs measuring between 15 and 20 mm, and furthermore, the last guidelines [8] cautiously suggest endoscopic resection for tumors <15 mm in selected cases only. Another possible limitation is that all the examined studies were retrospective case series; however, this is common in the neuroendocrine setting, due to the more indolent behavior of these tumors if compared to their exocrine counterparts, and they were overall moderate quality studies. Furthermore, some studies had a relatively short follow-up (i.e., less than 24 months), with a mean follow-up for all the included studies of 49 months. It is also important to highlight that our work might appear to be more aligned with a systematic review than a meta-analysis in the strict scientific sense. Surely, given the heterogeneity and limited sample size of the available studies, a comprehensive meta-analysis across all intended metrics was quite challenging. However, we have still performed a quantitative synthesis of the recurrence rates and safety results of the included studies, selecting only outcome measures where we did not find significant heterogeneity, which we believe allows current papers still to be considered as meta-analysis.

On the other hand, the greatest point of strength for our study is its systematic nature, providing an initial analysis of a niche but ambiguous topic that was not present in the literature yet and may pave the way to further studies whenever more data become available.

In conclusion, endoscopic resection could be the first treatment option in patients with dNENs sized 10–20 mm and without evidence of metastatic disease. However, the results of the current study must be interpreted with caution considering the intrinsic limitations of the included studies (i.e., the retrospective nature, the moderate scientific quality, the small sample size, and the short follow-up periods). Further studies are needed to better clarify the role of endoscopic resection in this subset of tumors and to provide evidence of its superiority among the available endoscopic techniques. In this specific setting, even more than in other NENs, the multidisciplinary management of the patients together with their referral to tertiary centers with higher endoscopic expertise is pivotal.

## Figures and Tables

**Table 1:** Summary of the included studies.

Authors	Shroff [12] *	Massironi [5] *	Lee [13] *	Tran [14] *	Mahmud [15] *	Yokoyama [16] *	Gincul [17] *
Number of total patients	30	108	60	104	33	4	29
Number of patients with 10–20 mm dNENs	4	8	9	20	9	4	11
Median size (range), (mm)	13 (10–19)	11 (10–14)	NA	13 (10–16)	12 (11–15)	12 (11–13)	NA
G1	NA	6	7	8	NA	NA	NA
G2	NA	1	2	5	NA	NA	NA
G3	NA	0	0	0	NA	NA	NA
EMR	4	7	5	20	8	4	11
ESD	0	1	4	0	0	0	0
Snare polipectomy	0	NA	0	0	1	0	0
En Bloc	NA	NA	8	NA	7	4	NA
Piecemeal	NA	NA	1	NA	2	0	NA
R0	2	NA	0	NA	5	4	4
R1	2	NA	9	NA	4	0	5

dNENs: duodenal neuroendocrine neoplasms; EMR: endoscopic mucosal resection; ESD: endoscopic submucosal dissection; NA: not available; R0: free margin after endoscopic resection; R1: positive margin after endoscopic resection. Grading was determined according to the WHO 2010 classification system. * Number in brackets represent the corresponding reference number.

## References

[1] Yao J.C., Hassan M., Phan A., Dagohoy C., Leary C., Mares J.E., Abdalla E.K., Fleming J.B., Vauthey J.N., Rashid A. (2008). One hundred years after “carcinoid”: Epidemiology of and prognostic factors for neuroendocrine tumors in 35,825 cases in the United States. J. Clin. Oncol..

[2] O’connor J.M., Marmissolle F., Bestani C., Pesce V., Belli S., Dominichini E., Mendez G., Price P., Giacomi N., Pairola A. (2014). Observational study of patients with gastroenteropancreatic and bronchial neuroendocrine tumors in Argentina: Results from the large database of a multidisciplinary group clinical multicenter study. Mol. Clin. Oncol..

[3] Dasari A., Shen C., Halperin D., Zhao B., Zhou S., Xu Y., Shih T., Yao J.C. (2017). Trends in the Incidence, Prevalence, and Survival Outcomes in Patients with Neuroendocrine Tumors in the United States. JAMA Oncol..

[4] Niederle M.B., Hackl M., Kaserer K., Niederle B. (2010). Gastroenteropancreatic neuroendocrine tumours: The current incidence and staging based on the WHO and European Neuroendocrine Tumour Society classification: An analysis based on prospectively collected parameters. Endocr. Relat. Cancer.

[5] Massironi S., Campana D., Partelli S., Panzuto F., Rossi R.E., Faggiano A., Brighi N., Falconi M., Rinzivillo M., Fave G.D. (2018). Heterogeneity of Duodenal Neuroendocrine Tumors: An Italian Multi-center Experience. Ann. Surg. Oncol..

[6] Gamboa A.C., Liu Y., Lee R.M., Zaidi M.Y., Staley C.A., Kooby D.A., Winer J.H., Shah M.M., Russell M.C., Cardona K. (2019). Duodenal neuroendocrine tumors: Somewhere between the pancreas and small bowel?. J. Surg. Oncol..

[7] Delle Fave G., O’Toole D., Sundin A., Taal B., Ferolla P., Ramage J.K., Ferone D., Ito T., Weber W., Zheng-Pei Z. (2016). ENETS Consensus Guidelines Update for Gastroduodenal Neuroendocrine Neoplasms. Neuroendocrinology.

[8] Panzuto F., Ramage J., Pritchard D.M., van Velthuysen M.F., Schrader J., Begum N., Sundin A., Falconi M., O’Toole D. (2023). European Neuroendocrine Tumor Society (ENETS) 2023 guidance paper for gastroduodenal neuroendocrine tumours (NETs) G1–G3. J. Neuroendocr..

[9] Rossi R.E., Milanetto A.C., Andreasi V., Campana D., Coppa J., Nappo G., Rinzivillo M., Invernizzi P., Modica R., David A. (2021). Risk of preoperative understaging of duodenal neuroendocrine neoplasms: A plea for caution in the treatment strategy. J. Endocrinol. Investig..

[10] Esposito G., Dell’unto E., Ligato I., Marasco M., Panzuto F. (2023). The meaning of R1 resection after endoscopic removal of gastric, duodenal and rectal neuroendocrine tumors. Expert Rev. Gastroenterol. Hepatol..

[11] Lijmer J.G., Mol B.W., Heisterkamp S., Bonsel G.J., Prins M.H., van der Meulen J.H.P., Bossuyt P.M.M. (1999). Empirical Evidence of Design-Related Bias in Studies of Diagnostic Tests. JAMA.

[12] Shroff S.R., Kushnir V.M., Wani S.B., Gupta N., Jonnalagadda S.S., Murad F., Early D.S., Mullady D.K., Edmundowicz S.A., Azar R.R. (2015). Efficacy of Endoscopic Mucosal Resection for Management of Small Duodenal Neuroendocrine Tumors. Surg. Laparosc. Endosc. Percutan. Tech..

[13] Lee S.W., Sung J.K., Cho Y.S., Bang K.B., Kang S.H., Kim K.B., Kim S.H., Moon H.S., Song K.H., Kim S.M. (2019). Comparisons of therapeutic outcomes in patients with nonampullary duodenal neuroendocrine tumors (NADNETs): A multicenter retrospective study. Medicine.

[14] Tran C.G., Sherman S.K., Suraju M.O., Nayyar A., Gerke H., El Abiad R.G., Chandrasekharan C., Ear P.H., O’dorisio T.M., Dillon J.S. (2022). Management of Duodenal Neuroendocrine Tumors: Surgical versus Endoscopic Mucosal Resection. Ann. Surg. Oncol..

[15] Mahmud N., Tomizawa Y., Stashek K., Katona B.W., Ginsberg G.G., Metz D.C. (2019). Endoscopic Resection of Duodenal Carcinoid Tumors: A Single-Center Comparison between Simple Polypectomy and Endoscopic Mucosal Resection. Pancreas.

[16] Yokoyama S., Takifuji K., Tani M., Kawai M., Naka T., Uchiyama K., Yamaue H. (2011). Endoscopic resection of duodenal bulb neuroendocrine tumor larger than 10 mm in diameter. BMC Gastroenterol..

[17] Gincul R., Ponchon T., Napoleon B., Scoazec J.-Y., Guillaud O., Saurin J.-C., Ciocirlan M., Lepilliez V., Pioche M., Lefort C. (2016). Endoscopic treatment of sporadic small duodenal and ampullary neuroendocrine tumors. Endoscopy.

[18] Iwasaki T., Nara S., Kishi Y., Esaki M., Shimada K., Hiraoka N. (2017). Surgical treatment of neuroendocrine tumors in the second portion of the duodenum: A single center experience and systematic review of the literature. Langenbeck’s Arch. Surg..

[19] Clark O.H., Ajani J.A., Benson A.B., Berlin J.D., Blaszkowsky L.S., Byrd D., Choti M.A., Doherty G.M., Engstrom P.F., Gibbs J.F. (2009). NCCN Clinical Practice Guidelines in Oncology: Neuroendocrine tumors. J. Natl. Compr. Cancer Netw..

[20] Varas M.J., Gornals J.B., Pons C., Espinós C., Abad R., Lorente F.J., Bargalló D. (2010). Usefulness of endoscopic ultrasonography (EUS) for selecting carcinoid tumors as candidates to endoscopic resection. Rev. Esp. Enferm. Dig..

[21] Kim G.H., Kim J.I., Jeon S.W., Moon J.S., Chung I.K., Jee S.R., Kim H.U., Seo G.S., Baik G.H., Lee Y.C. (2014). Endoscopic resection for duodenal carcinoid tumors: A multicenter, retrospective study. J. Gastroenterol. Hepatol..

[22] Alexander S., Bourke M.J., Williams S.J., Bailey A., Co J. (2009). EMR of large, sessile, sporadic nonampullary duodenal adenomas: Technical aspects and long-term outcome (with videos). Gastrointest. Endosc..

[23] Brito H.P., Torres I.T., Turke K.C., Parada A.A., Waisberg J., Botelho R.V. (2021). Comparison of endoscopic resection techniques for duodenal neuroendocrine tumors: Systematic review. Endosc. Int. Open.

[24] Suzuki S., Ishii N., Uemura M., Deshpande G.A., Matsuda M., Iizuka Y., Fukuda K., Suzuki K., Fujita Y. (2012). Endoscopic submucosal dissection (ESD) for gastrointestinal carcinoid tumors. Surg. Endosc..

[25] Honda T., Yamamoto H., Osawa H., Yoshizawa M., Nakano H., Sunada K., Hanatsuka K., Sugano K. (2009). Endoscopic submucosal dissection for superficial duodenal neoplasms. Dig. Endosc..

[26] Margonis G.A., Samaha M., Kim Y., Postlewait L.M.L., Kunz P., Maithel S.K., Poultsides G.A., Tran T., Gamblin T.C., Berger N.G. (2016). A Multi-institutional Analysis of Duodenal Neuroendocrine Tumors: Tumor Biology Rather than Extent of Resection Dictates Prognosis. J. Gastrointest. Surg..

[27] Ragheb J., Mony S., Klapman J., Erim T., Reagan A., Butler R., Dong Y., Cruise M., Centeno B.A., Bejarano P. (2021). Impact of margin status on outcomes after endoscopic resection of well-differentiated duodenal neuroendocrine tumors. Gastrointest. Endosc..

